# Quincke's Triad and Cystic Artery Pseudoaneurysm

**DOI:** 10.7759/cureus.77627

**Published:** 2025-01-18

**Authors:** Mohamed Ahmed, Rasha Saeed, Carson Woodward, Kim Nguyen, Danya Auda

**Affiliations:** 1 Surgery, University of California, Riverside, Riverside, USA; 2 Surgery, AdventHealth - Tampa, Tampa, USA; 3 Occupational Medicine/Environmental medicine, University of California, Irvine, Irvine, USA; 4 Psychology, University of California, Riverside, Riverside, USA

**Keywords:** chronic abdominal pain, chronic calculous cholecystitis, gastrointestinal bleeding, haemobilia, splanchnic artery pseudo aneurysm coiling and embolization

## Abstract

Cystic artery pseudoaneurysms (CAP) are rare and occur as a result of chronic inflammatory conditions or trauma including a difficult laparoscopic cholecystectomy. We present a case of a 66-year-old female who presented to our emergency room with a two-day history of abdominal pain, jaundice, and melena, symptoms which were retrospectively identified as components of Quincke's triad. After an initial endoscopic retrograde cholangiopancreatography (ERCP), the patient underwent an attempted laparoscopic cholecystectomy complicated by massive bleeding requiring conversion to attempted open cholecystectomy, and damage control surgery. After angioembolization of the cystic artery, the patient returned to the operating room and cholecystectomy was performed. This case highlights this rare presentation where unplanned initial management can result in life-threatening consequences.

## Introduction

Cystic artery pseudoaneurysms (CAPs) account for less than 1% of all arterial aneurysms; they are the result of trauma or intra-abdominal inflammatory processes that have been known to present with Quincke's clinical triad [1]. It has also been reported that hepatocellular carcinoma ruptures in the gallbladder cause CAP and haemobilia [2]. A high index of suspension is needed to establish Quincke’s triad; jaundice, right upper quadrant abdominal pain, and upper gastrointestinal bleeding are present in approximately only 35-40% of cases [3]. Trauma is the most common cause of haemobilia, accounting for up to 85% of cases [4].

## Case presentation

**Figure 1:**
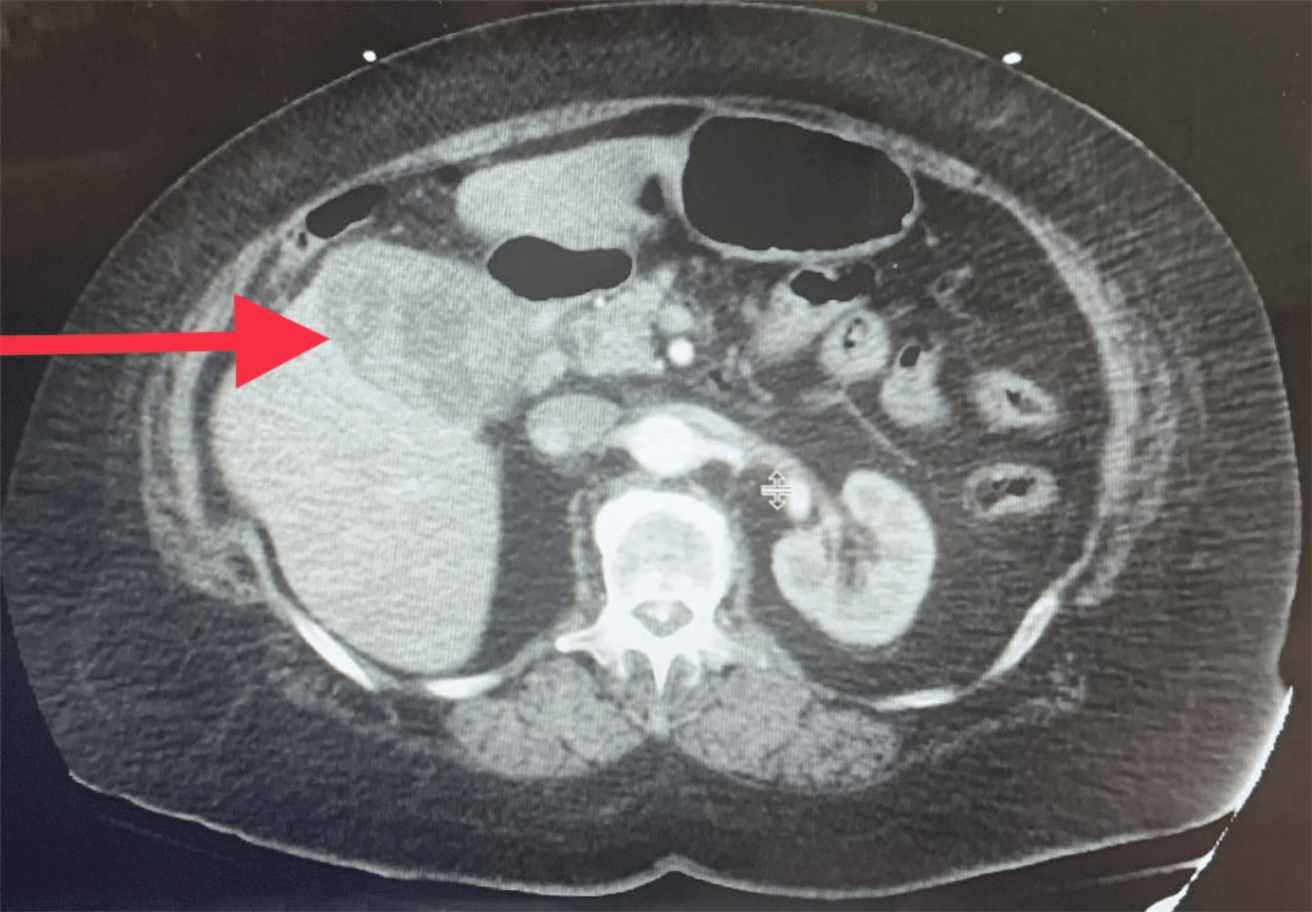
Computerized tomography of the abdomen Thickened gallbladder wall concerning underlying malignancy (red arrow).

**Figure 2:**
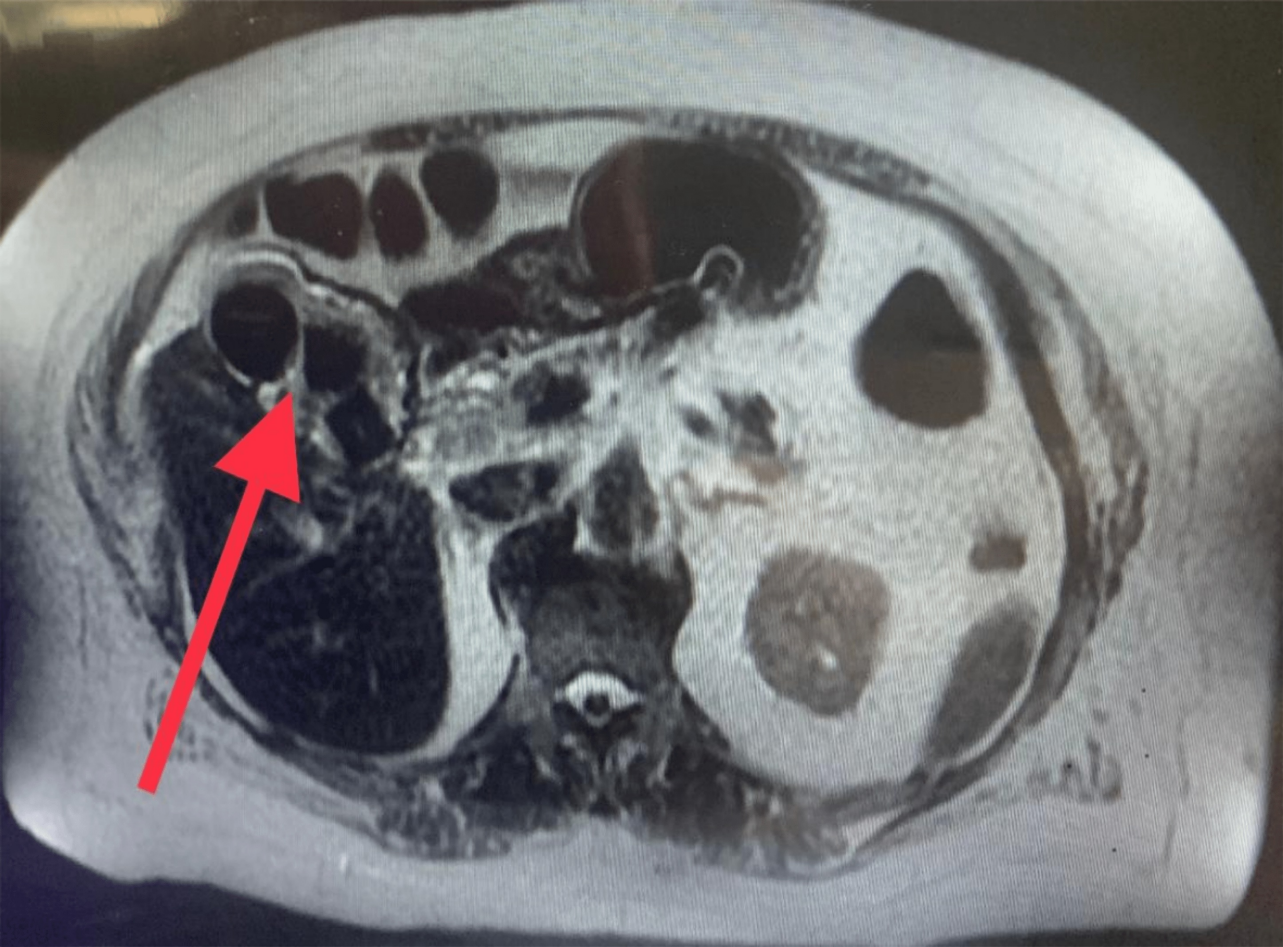
Magnetic resonance imaging of the abdomen. Inflamed gallbladder with large stones (red arrow).

A 66-year-old female patient presented with worsening right upper quadrant pain associated with nausea and vomiting over the last two days. The patient had chills and a dark foul-smelling stool which prompted her to come to the emergency room. She had a nerve stimulator for chronic back pain removed two weeks prior and has been using non-steroidal anti-inflammatory drugs and narcotics to control her symptoms. Physical evaluation revealed right upper quadrant tenderness. Laboratory findings total bilirubin 7.5 mg/dl (reference 0-1.1 mg/dl), direct bilirubin 5.6 mg/dl (reference 0.0-0.30 mg/dl), alkaline phosphatase 279 U/L (reference 26-137 U/L), AST 54 U/L (reference 0-37 U/L), ALT 191 U/L (reference 0-60 U/L), hemoglobin 12.6 g/dl (reference 11.7-15.5 gm/dl). Computerized tomography (CT) revealed a thickened gallbladder wall due to inflammation and cannot rule out malignancy (Figure 1). While the patient’s symptoms were consistent with Quincke's triad, this was initially overlooked at presentation and only retrospectively identified. Magnetic resonance imaging revealed an inflamed gallbladder containing large stones with no biliary duct obstruction (Figure 2).

**Figure 3:**
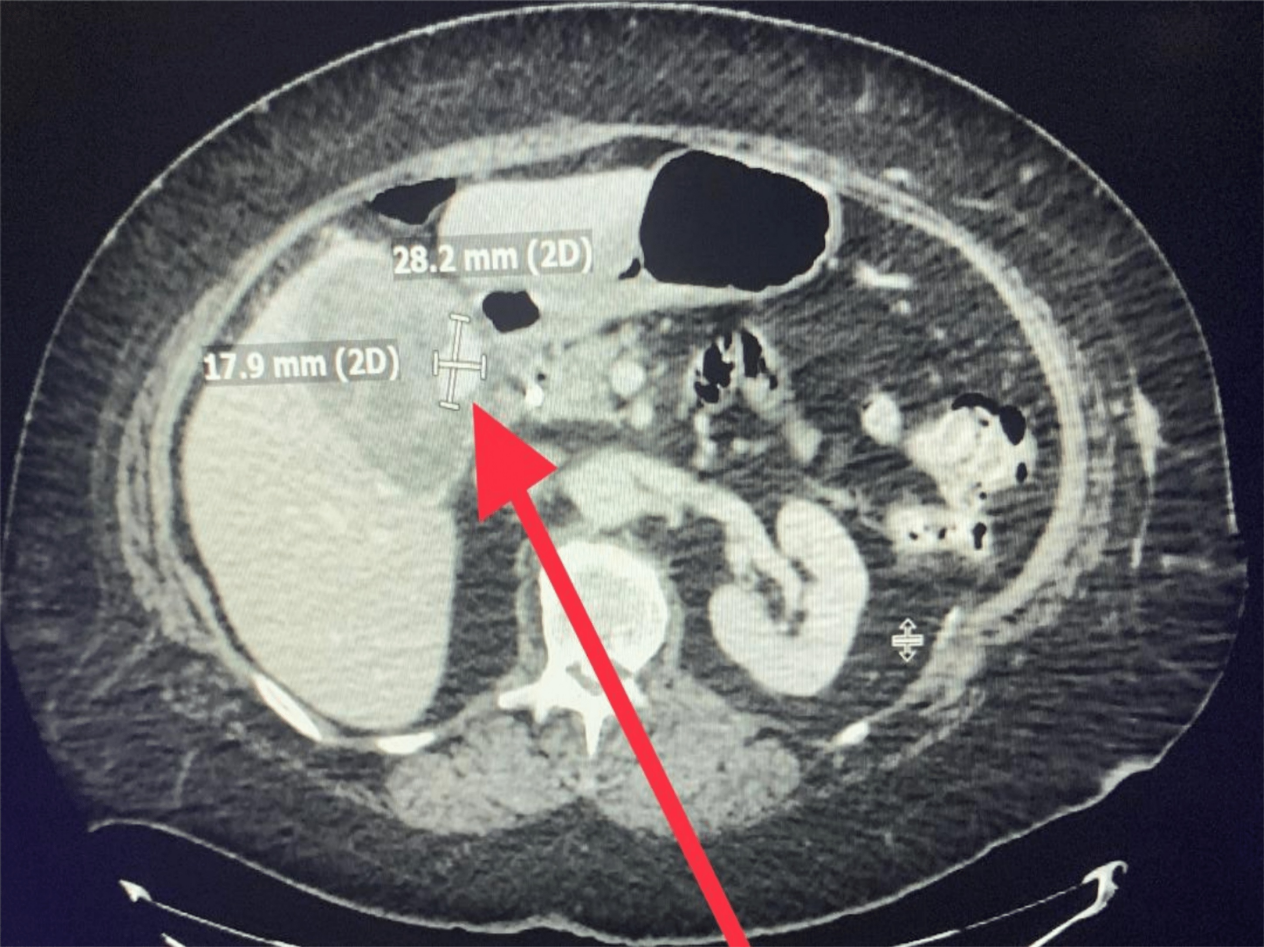
Computerized tomography of the abdomen with arterial contrast. The pseudoaneurysm is indicated by the red arrow.

Upper endoscopy and endoscopic retrograde cholangiopancreatography (ERCP) revealed blood clots and thick mucoid sludge in the common bile duct. The patient's liver function test trended down, with a decrease in haemoglobin levels to 9.8 g/dl, associated with melena. Repeated ERCP with stenting was performed three days after the first procedure with the evacuation of blood clots from the common bile duct. CT abdomen with arteriography was performed and a 2.8 x 1.7 cm nodular contrast focus in the gallbladder was consistent with pseudoaneurysm (Figure 3).

**Figure 4:**
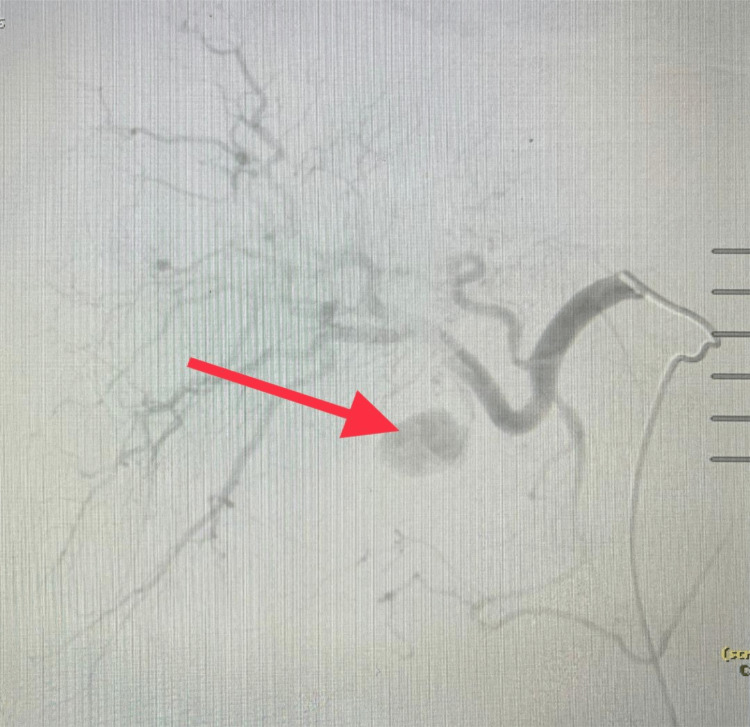
Hepatic artery angiography. Cystic artery pseudoaneurysm (red arrow).

**Figure 5:**
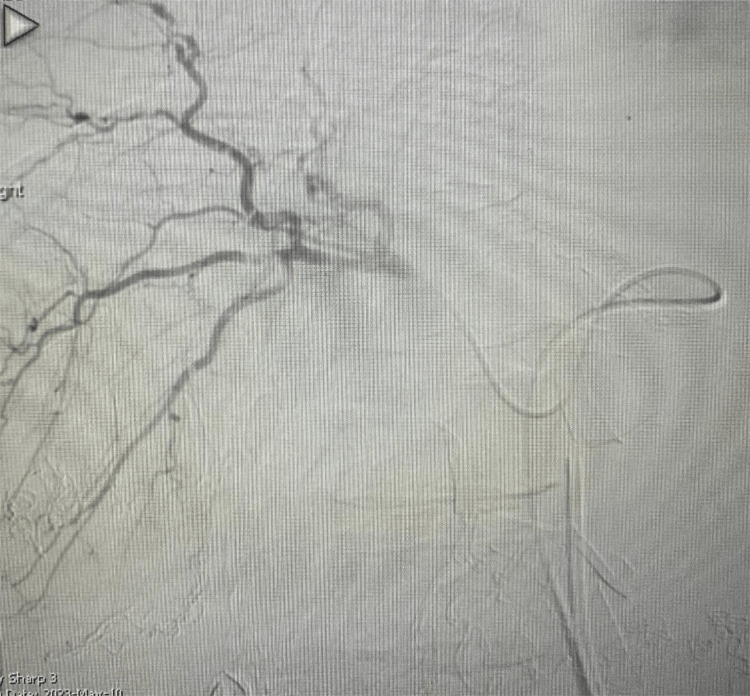
Hepatic artery arteriography after gel foam and thrombin embolization of the cystic artery No further active bleeding was seen.

The patient underwent an attempted laparoscopic cholecystectomy which was complicated by massive active bleeding from a torn gallbladder caused due to attempts to retract it; a laparotomy was performed and the gallbladder was very friable. The resulting bleeding was difficult to control despite manual control of the portal triad. Multiple large stones were removed, and the gallbladder was packed with SurgiSeal. Liver packing was performed, and the temporary dressing was applied. The patient was transferred to the interventional radiology (IR) suite. Embolization of the cystic artery with gel foam and thrombin controlled the active bleeding (Figures 4-5).

The patient returned to the operating room the following day, and packing removal, cholecystectomy, drain placement, and abdomen closure were performed. The patient did well and was discharged to a skilled nursing facility four days later.

## Discussion

Haemobilia, defined as bleeding from or into the biliary tree, was first defined by Sandblom in 1948 [5]. An aneurysm is an excessive enlargement of an artery as a result of arterial wall weakness. While true aneurysms maintain all components of the arterial wall, pseudoaneurysm does not and it occurs within the adventitial layer after an intimal injury [6]. Though there are only a few reported cases in the literature, CAP is a life-threatening rare pathology as a result of cholecystitis (61.2%), post-cholecystectomy (26.8%), cholelithiasis (1.5%), idiopathic causes, which account for (8.9%) of cases, and pancreatitis (1.5%) [7]. The duration of CAP rupture can range from eight days to three years and is associated with high mortality (21-43%) [8]. CAP diagnosis can be very challenging clinically as most patients initially present with vomiting, nausea, melena, vague abdominal pain, and fever, which can represent a wider differential diagnosis as only 40% of patients present with all three symptoms of the classic Quincke's triad, upper gastrointestinal bleeding (45%), jaundice (60%), and right upper quadrant abdominal pain (70%) [9]. 

The median age of diagnosis for CAP is 68 years and the majority of reported cases involved older patients with comorbidities, including the presence of atherosclerosis, diabetes, hypertension, hypercholesterolemia, and vasculitis [10]. Hemorrhagic shock as a result of ruptured CAP is associated with close to 50% mortality rate [11,12]. Computerized tomo-angiography is the most sensitive imaging method for diagnoses [13]. Angioembolization as a treatment was first described by Walter in 1978 and has since become the mainstay of treatment in most cases [14]. Percutaneous thrombin injection of CAP has been reported as an option [15]. Surgery can be a treatment modality in planned cases and when angioembolization is not an option [16].

## Conclusions

CAP is rare, accounting for less than 1% of arterial aneurysms caused by trauma or intra-abdominal inflammatory processes. They most commonly present with hematobilia, which can be difficult to diagnose. A high index of suspicion and prompt targeted imaging and intervention is required. Arterial embolization before cholecystectomy is recommended to avoid life-threatening situations.
